# Epithelial Cell Gene Expression Induced by Intracellular *Staphylococcus aureus*

**DOI:** 10.1155/2009/753278

**Published:** 2009-02-03

**Authors:** Xianglu Li, William G. Fusco, Keun S. Seo, Kenneth W. Bayles, Erin E. Mosley, Mark A. McGuire, Gregory A. Bohach

**Affiliations:** ^1^Department of Microbiology, Molecular Biology and Biochemistry, University of Idaho, Moscow, ID 83844, USA; ^2^Environmental Biotechnology Institute, University of Idaho, Moscow, ID 83844, USA; ^3^Department of Pathology & Microbiology, University of Nebraska Medical Center, Omaha, NE 68198, USA; ^4^Department of Animal and Veterinary Science (AVS), University of Idaho, Moscow, ID 83844, USA

## Abstract

HEp-2 cell monolayers were cocultured with intracellular *Staphylococcus aureus*, and changes in gene expression were profiled using DNA microarrays. Intracellular *S. aureus* affected genes involved in cellular stress responses, signal transduction, inflammation, apoptosis, fibrosis, and cholesterol biosynthesis. Transcription of stress response and signal transduction-related genes including *atf3, sgk, map2k1, map2k3, arhb*, and *arhe* was increased. In addition, elevated transcription of proinflammatory genes was observed for *tnfa, il1b, il6, il8, cxcl1, ccl20, cox2,* and *pai1*. Genes involved in proapoptosis and fibrosis were also affected at transcriptional level by intracellular *S. aureus*. Notably, intracellular *S. aureus* induced strong transcriptional down-regulation of several cholesterol biosynthesis genes. These results suggest that epithelial cells respond to intracellular *S. aureus* by inducing genes affecting immunity and in repairing damage caused by the organism, and are consistent with the possibility that the organism exploits an intracellular environment to subvert host immunity and promote colonization.

## 1. Introduction

*Staphylococcus aureus* (*S. aureus*), a nosocomial or community-acquired pathogen that colonizes much of the healthy population [1], is an important cause of skin infections, pneumonia, septicemia, endocarditis, osteomyelitis, folliculitis, mastitis, and other infections. The organism also causes toxigenic illnesses such as food poisoning and toxic shock syndrome [2]. Infections caused by *S. aureus* may be refractory to therapy and become chronic or recur, despite acceptable therapy [3–6].

Several studies showed that *S. aureus* can become internalized by nonprofessional phagocytes [7–9]; *α*_5_ *β*_1_ integrin is necessary for fibronectin-mediated *S. aureus* internalization involving staphylococcal fibronectin-binding proteins [10, 11]. Internalization may provide several benefits to *S. aureus*. It has been proposed that intracellular *S. aureus* evades exposure to antibiotics [3] and host immunity. It also provides an intracellular milieu which leads to the formation of small-colony variants with decreased metabolic activity and increased antibiotic resistance [12].

Microarray technology has helped elucidate pathogen-host cell interactions and profile the effects on epithelial cells by organisms including, but not limited to, *Yersinia enterocolitica* [13], *Salmonella dublin* [14], *Shigella flexneri* [15], *Bordetella pertussis* [16], *Mycobacterium tuberculosis* [17], *Pseudomonas aeruginosa* [18], *Listeria monocytogenes* [19], *Streptococcus pyogenes* [20], and *S. aureus* [21, 22]. Although the internalization of *S. aureus* by nonprofessional phagocytes is well documented [5, 7–9, 23–25], the cellular response to intracellular *S. aureus* has only been partially elucidated [3, 26], focusing mainly on apoptosis [27–33]. The present study assessed global changes in gene expression over an 8-hour time period in epithelial cell monolayers induced by intracellular *S. aureus*. The data demonstrated that cultured epithelial cells respond to intracellular *S. aureus* by inducing several classes of genes that could influence the outcome of colonization or infection by this organism in vivo.

## 2. Materials and Methods

### 2.1. Cultures

HEp-2 cells [34] were purchased from the American Type Culture Collection (ATCC). Routine maintenance was conducted using complete growth medium (CGM) [10]. *S. aureus* RN6390 [32, 33, 35] provided by A. Cheung (Dartmouth Medical School) was used to infect HEp-2 cells using established techniques described previously [8, 32, 33, 36]. Briefly, bacteria from 16-hour Todd Hewitt broth cultures were washed three times with phosphate buffered saline (PBS), and resuspended in invasion medium (IM; CGM lacking antibiotics and FBS) to make stocks with approximately 10^9^ colony-forming units (CFU) mL^−1^. Bacterial stocks were diluted 10-fold in fresh IM; 500 *μ*L of the cell suspension well^−1^ were used to infect each HEp-2 culture at a multiplicity of infection (MOI) of 10. The cocultures were centrifuged immediately to synchronize monolayer infections and incubated at 37°C for 10 minutes to allow internalization, after 10 minutes, the IM was rapidly replaced with fresh medium containing gentamicin (100  *μ*g mL^−1^) to kill noninternalized bacteria. Thereafter, the cocultures were incubated (up to 8 hours following *S. aureus* exposure) and analyzed at various times following exposure to *S. aureus* as described below.

For growth rate analyses, cells from 16-hour *S. aureus* RN6390 TH broth cultures (above) were pelleted, washed three times with PBS, and diluted with PBS to 10^5^ CFU mL^−1^. A 100 *μ*L aliquot was inoculated into 10 mL of TH broth or IM, with or without FBS (without antibiotics). Cultures were incubated with vigorous shaking up to 8 hours. CFU concentrations were determined by a standard plate count method.

### 2.2. RNA Isolation and Purification

HEp-2 cells were harvested at 2, 4, 6, or 8 hours following addition of bacteria. RNA was isolated using TRIZOL (Invitrogen) according to the manufacturer's instructions and further purified with RNAeasy MinElute Cleanup Kits (Qiagen). RNA samples, quantified using a NanoDrop ND-1000 spectrophotometer (Nanodrop Technologies) and showing OD_260_:OD_280_ ratios >1.95 were used for subsequent experiments.

### 2.3. Microarray Methods and Data Analysis

MWG Human 30K microarrays (MWG) were used according to the manufacturer's instructions. cDNA was synthesized using the BD Atlas PowerScript Fluorescent Labeling Kit (BD) with oligo(dT)_12-18_ primer (Invitrogen). CyDye Post-Labeling Reactive Dyes (Amersham) were used to fluorescently label the cDNA (Cy3 for cDNA from uninfected cells and Cy5 for cDNA from *S. aureus* infected cells). Unincorporated dye was removed from labeled cDNA with CHROMA SPIN+TE-30 columns (Clontech). Labeled cDNA was dissolved in salt-based hybridization buffer (MWG), incubated at 95°C (3 minutes), chilled on ice, and hybridized to the microarray chips in the dark for 16–24 hours at 42°C with slow rocking. Arrays were washed and scanned with an Axon 4000A dual channel microarray scanner (Axon) to generate multi-TIFF images which were processed with GenePix Pro 6.0 software (Molecular Devices).

### 2.4. Quantitative Real-Time PCR (QRT-PCR)

QRT-PCR was used to validate selected microarray data. cDNA was synthesized from 1 *μ*g of RNA using Superscript Π Reverse Transcriptase (Invitrogen). Primers (Table 1), designed using Primer Express 2.0 software (PE Applied Biosystems), were purchased from Integrated DNA Technologies (IDT). Data were analyzed as described previously [37]. The threshold cycle (*C*_*T*_) was calculated as the cycle number at which the ΔRn crossed the baseline. Data were normalized by calculating Δ*C*_*T*_ [*C*_*T*_ of target − *C*_*T*_ of the internal control (*β*-actin)]. Normalized Δ*C*_*T*_ data from *S. aureus* infected HEp-2 cells were compared to data from uninfected HEp-2 cells by calculating ΔΔ*C*_*T*_ [Δ*C*_*T*_ of *S. aureus* infected HEp-2 cells − Δ*C*_*T*_ of uninfected HEp-2 cells]. Each experiment was conducted thrice for validation, and the mean value is reported.

### 2.5. Cholesterol Analyses

HEp-2 cells were dislodged with TrypLE Express (Gibco) and collected by centrifugation. Lipids were extracted with chloroform and methanol [38], analyzed and quantified by gas chromatography/mass spectrometry (GC-MS 6890N; Agilent Technologies) and reported as *μ*g/10^5^ cells. Each experiment was conducted at least three times.

### 2.6. Flow Cytometry

Prior to infection, *S. aureus* was labeled with 0.5 *μ*M 5- (and-6)-carboxyfluorescein diacetate, succinimidyl ester (CFSE) (Invitrogen) for 10 minutes at 37°C. CFSE-stained *S. aureus* was washed three times with PBS and used to infect HEp-2 cells as described above. After coculturing for 10 minutes, cells were washed and incubated (15 minutes, 37°C) with *S. aureus* specific antibody ab37644 (Abcam), followed by goat antimouse IgG conjugated with Cy5 (Southern Biotech) to quantify extracellular bacteria. In parallel experiments to quantify extracellular bacteria, infected monolayers were treated with lysostaphin for 2 hours resulting in loss of the CFSE signal. Confirmation of the effectiveness of lysostaphin treatment was accomplished by treatment with Cy5-conjugated antibody as described above. Cells were harvested and analyzed with a FACSAria flow cytometer (BD), equipped with FACSDiva software (BD).

### 2.7. Statistical Analyses

GeneSpring version 7.2 (Silicon Genetics) was used to analyze microarray data. For each time point, data from 3–5 separate replicated experiments were obtained and analyzed by 2-way ANOVA (*P* < .05) to determine their validity, followed by Benjamini and Hochberg false discovery rate correction for each data set [39]. Correction for spot intensity variations among arrays was performed by intensity-dependent normalization and subtraction of background based on negative controls. Normalized mean values were determined for all data points. Microarray data were reported as increased or decreased expression (>1.0 or <1.0, resp.) by dividing the mean Cy5 value (infected HEp-2 cells) by the mean Cy3 value (uninfected HEp-2 cells) for each time point.

## 3. Results and Discussion

### 3.1. Experimental Model

**Figure 1:**
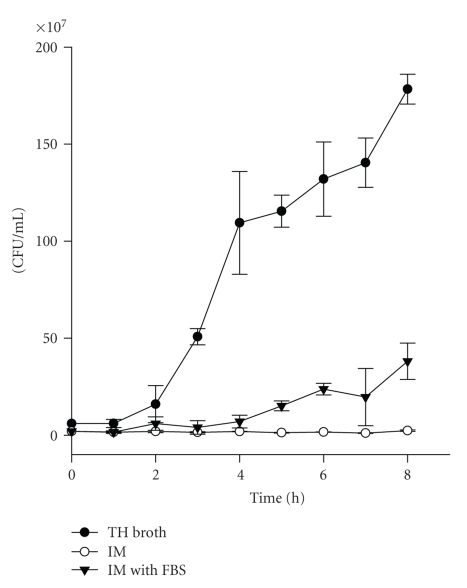
Growth analysis of *S. aureus* RN6390. To assess growth, *S. aureus* RN6390 was inoculated into different media (TH broth, IM, or IM supplemented with FBS). CFUs were determined hourly by a standard plate count method up to 8 hours, and represented as the mean ± SEM of data acquired from three experiments.

As this study was designed to assess the effects of internalized *S. aureus* on the HEp-2 pharyngeal epithelial cell line, the influences of extracellular bacteria or their exotoxins produced prior to internalization of *S. aureus* were minimized by (1) thoroughly washing the inocula; (2) treating cocultures with gentamicin after a very short (10 minutes) extracellular bacterial exposure; (3) conducting the extracellular exposure period in a medium that does not support extracellular growth. Specifically, unlike control cultures in TH broth which supported robust growth, *S. aureus* RN6390 cultured in IM did not grow, even when incubated for periods of time much longer than the 10 minutes used to infect cells (Figure 1). Furthermore, IM supplemented with FBS supported moderate growth, indicating that a lack of growth in IM alone was not due to inhibitory components.

**Figure 2:**
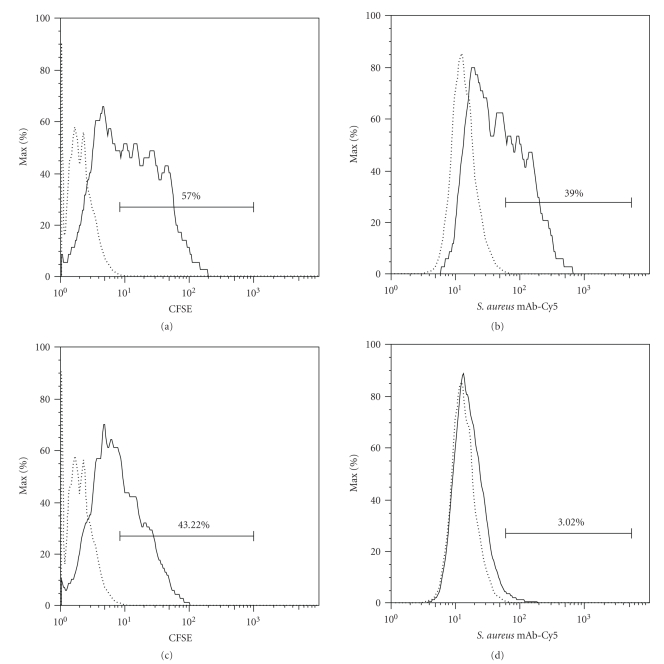
Assessment of *S. aureus* RN6390 internalization using flow cytometry. Dotted lines indicate the uninfected HEp-2 cell control, and solid lines indicate HEp-2 cells infected with CFSE-labeled *S. aureus* ((a) and (c)) or infected with CFSE-labeled *S. aureus* followed by labeling Cy5-conjugated mAb specific for *S. aureus* ((b) and (d)). In panels (a) and (b), HEp-2 cells were infected with CFSE-labeled *S. aureus* without treatment with lysostaphin. CFSE signal represents HEp-2 cells infected with extracellular and/or intracellular *S. aureus* (a). Cy5 signal represents HEp-2 cells infected with extracellular *S. aureus* only (b). In panels (c) and (d), HEp-2 cells were infected with CFSE-labeled *S. aureus* followed by the treatment with lysostaphin which degrades staphylococcal cell wall causing a loss of CFSE signal by extracellular *S. aureus*. CFSE signal represents HEp-2 cells infected with intracellular *S. aureus* only (c). This was confirmed by showing the loss of Cy5 signal in panel (d). Data shown are from a representative experiment which was conducted three times.

Considering the short exposure of HEp-2 cells to extracellular *S. aureus*, it was of interest to quantify the percentage of infected HEp-2 cells containing intracellular bacteria. This was accomplished by differential staining of intracellular and extracellular bacteria and by monitoring intracellular CFSE-stained *S. aureus* following lysostaphin treatment to remove extracellular bacteria. As shown in Figure 2(a), a 10-minute-exposure resulted in monolayers in which approximately 57.0% of the HEp-2 cells contained cell-associated *S.* *aureus* (extracellular and/or intracellular), while approximately 39.0% of HEp-2 cells were associated with extracellular bacteria (Figure 2(b)). Lysostaphin treatment which removed nearly all extracellular bacteria (Figure 2(d)) revealed that approximately 43.2% of the HEp-2 cells had intracellular *S. aureus* (Figure 2(c)).

### 3.2. Microarray and QRT-PCR Data Analysis

Intracellular *S. aureus* altered expression of several classes of HEp-2 genes. Genes with statistically validated altered transcription levels >1.50-fold (increase or decrease) at any of the four-time-points in microarrays are listed in Table 2. To avoid potential pitfalls associated with amplification of mRNA such as inferior reducibility, mRNA was not amplified in this study. The microarray data shown here represented true transcription levels. Although we suspect that relatively low mRNA levels resulted in microarray data for some samples which were not statistically significant (*P* > .05), data for selected genes of interest were validated by QRT-PCR (summarized in Table 3). Data not shown in Table 2 resulted from signal intensities <50 which were too low to quantify.

### 3.3. Stress Response

The adaptor-related protein complex 1 (AP-1) comprises JUN, FOS, and activating transcription factor (ATF) proteins; it regulates a variety of activities including proliferation, apoptosis, and inflammation in response to stress signals, cytokines, growth factors, and microbial infections [40, 41]. Internalization of *S. aureus* induced a rapid (7.89-fold) increase in *atf3* mRNA levels at 2 hours postinfection that rapidly declined thereafter, as measured by microarray analysis (Table 2). QRT-PCR analysis yielded consistent findings (Table 3). Other AP-1 genes, such as *c-fos*, *fosB*, *c-jun,* and *junB*, were up-regulated as measured by microarray and/or QRT-PCR analysis, albeit less dramatically at 2 hours. Another stress response gene, *sgk*, encoding serum and glucocorticoid-induced protein kinase (SGK) [42], was up-regulated maximally at 2 hours (Tables 2 and 3). SGK is involved in epithelial sodium transport, and is induced in epithelial cells in response to environmental stimuli and stress [42].

### 3.4. Signal Transduction

Intracellular *S. aureus* also affected genes involved in several mitogen-activated protein kinase (MAPK) pathways. MAPK kinase 1 (*map2k1*) mRNA levels gradually increased and reached a maximum level at 8 hours (Table 2), MAPK kinase 1 activates downstream extracellular signal-regulated protein kinases (ERKs) in the Ras-Raf-MEK-ERK pathway. Two Ras homolog genes, *arhe* and *arhb*, were generally up-regulated >1.50-fold throughout the 8-hour-infection (Tables 2 and 3), whereas, the Ras inhibitor gene, *ack-1*, was down-regulated (Table 2). Thus, up-regulation of *map2k1*, *arhe*, and *arhb,* and down-regulation of the inhibitor *ack-1* are consistent with activation of Ras-ERK pathway. Ras proteins are important for cytoskeleton reorganization [43, 44], coinciding with bacterial uptake and intracellular movement. Transcription of another MAPK gene (*map2k3*), a dual-specific kinase that phosphorylates MAPK14 (p38), was up-regulated >1.50-fold at all four-time-points (Tables 2 and 3). P38 pathway plays an important role in regulating proinflammatory gene expression including *tnfa*, *il1b*, and *cox2* [43, 44].

Staphylococcal activation of the ERK and P38 pathways in epithelial cells has also been observed in previous studies [45–47]. Activation of ERK and P38 pathways, in epithelial cells, was also seen in other intracellular pathogen infections such as *Helicobacter pylori* [48] and *Salmonella enterica* [49].

### 3.5. Proinflammatory Response

Intracellular bacteria frequently up-regulate several proinflammatory cytokine genes (*tnfa*, *il1b*, and *il6*) and chemokine genes (*il8*, *ccl20,* and *cxcl1*) [15, 49, 50]. Due to the low transcriptional activity of *il1b*, *tnfa*, *il6*, *cxcl1,* and *ccl20* in uninfected HEp2-cells, accurate comparison of these genes was not obtained with microarray analysis. QRT-PCR analysis demonstrated that transcription of *il1b*, *tnfa*, *il6*, *cxcl1,* and *ccl20* genes was up-regulated (Table 3), although only small to moderate increases were observed, compared to previous study [22]. This finding is likely due to differences in types of host cells and in *S. aureus* strains, and also due to the fact that we investigated only the effects of intracellular staphylococci. For example, human umbilical endothelial cells infected with a clinical *S. aureus* isolate, were induced expression of several proinflammatory cytokines/chemokines with similar fold changes to our study at transcriptional level. However, it did not induce expression of either *tnfa*, or *ilb*, which was different from our study [26]. Similarly, vaginal epithelial cells cocultured simultaneously with intracellular and extracellular *S. aureus* MNSM, producing toxic shock syndrome toxin-1, for 3 hours showed increases in the transcription of *il8*, *cxcl1*, and *ccl20* (11.3-fold, 17.1-fold and 207.9-fold, resp.) which were much stronger than our results [22].

Cyclooxygenase-2 gene (*cox2*), an inducible form of the cyclooxygenase-1 gene (*cox1*), was up-regulated at all four-time-points in this study (Tables 2 and 3). As an immediate early response gene that is responsible for prostanoid biosynthesis involved in proinflammation, *cox2* is expressed in epithelial cells, macrophages, fibroblasts, and vascular endothelial cells [51]. COX2 is induced by IL-1*β* [52] and lipoteichoic acid from *S. aureus* [53]. Up-regulation of *cox2* transcription was also associated with infection of epithelial cells by gram-negative bacteria: *Y. enterocolitica* [13] and *S. flexneri* M90T, probably via LPS [15]. The induction of *cox2* expression is not significantly in vaginal epithelial cell cultures infected (intracellular plus extracellular) with the superantigen producing strain *S. aureus* MNSM (see above) [22], further emphasizing the potentially different effects caused by various *S. aureus* strains, as well as the systems employed to measure their effects.

### 3.6. Cell Proliferation and Proapoptosis

Intracellular *S. aureus* RN6390 affected transcription of several proapoptotic genes. Dickkopf-1 (*dkk1*), was up-regulated >2.00-fold at all time points examined (Tables 2 and 3). Krüppel-like factors 4 and 6 genes (*klf4* and *klf6*) were up-regulated >2.00-fold at 2 hours postinfection (Table 2). Microarray data showed the gene for caspase-9 (*casp9*) up-regulated ~2.00-fold at 2 hours (Table 2), and this result was confirmed by QRT-PCR (Table 3). The gene (*bnip3*) encoding Bcl2/adenovirus E1B 19kDa interacting protein 3, a mitochondrial proapoptotic protein, was up-regulated >2.00-fold at both 6 hours and 8 hours (Table 2). Two insulin-like growth factor binding protein genes (*igfbp1* and *igfbp3*) were up-regulated >1.50-fold at 4 hours, 6 hours, and 8 hours postinfection (Tables 2 and 3). The NR4A1 receptor gene (*nur77*), which encodes a transcription factor that exhibits proapoptotic properties in T cells [54], was up-regulated ~6-fold at 2 hours (Table 2). These findings were similar to several studies demonstrating that the infection of epithelial cells [8, 28, 32, 33], endothelial cells [29, 30, 55, 56], and osteoblasts [3, 57, 58] with *S. aureus* can lead to apoptosis. Previous work in our lab had shown the involvement of host caspases 3 and 8 in *S. aureus*-induced apoptosis [32] and the requirement of the *S. aureus* virulence gene regulator *agr* in the induction of epithelial cell apoptosis [33].

### 3.7. Profibrotic Gene Transcription in HEp-2 Cells

TGF*β*1 is a key protein involved in many cell functions including fibrosis formation, regulation of cell cycle, apoptosis, and matrix remodeling [59]. QRT-PCR indicated that *tgf*β*1* was up-regulated by intracellular *S. aureus* (Table 3). Intracellular *S. aureus* also induced transcription of several genes related to TGF*β*1, especially in regard to fibrosis formation (Tables 2 and 3). In microarray experiments, transforming growth factor beta receptor 2 gene (*tgf*β*r2*) and epidermal growth factor receptor (EGFR) gene (*v-erb-b*) were up-regulated >1.5-fold after 4 hours (Table 2). Integrin *α*5 gene (*itga5*) was gradually up-regulated after 2-hour-infection (Table 2). The gene (*thbs1*) encoding thrombospondin 1 was up-regulated ~3.00-fold at 4 hours and 2.54-fold at 6 hours in microarray experiments (Table 2), and similarly, with QRT-PCR (Table 3).

Plasminogen activator inhibitor 1 and 2 genes (*pai1, pai2*) were up-regulated in microarray experiments (Table 2). Studies have shown that TGF*β*1 induces plasminogen activator inhibitor 1 (PAI1) expression and demonstrated the requirement for EGFR in this process [60–62]. Both PAI1 and PAI2 are inhibitors of the fibrinolysis system, acting to block the activity of tissue plasminogen activator and urokinase, and preventing the conversion of plasminogen to plasmin. Plasmin is a serine protease that degrades fibrin clots as well as extracellular matrix components. Thus, up-regulation of *pai1* and *pai2* may reduce extracellular matrix degradation.

The CCN (Cysteine-rich 61, Connective tissue growth factor, and Nephroblastoma overexpressed) family members are cysteine-rich and functionally diverse proteins that are involved in mitosis, apoptosis, adhesion, extracellular matrix production, angiogenesis, and tumor growth [63]. Three genes belonging to the CCN family were up-regulated. Two of those, *cyr61* and *ctgf*, were significantly up-regulated at early time points (Table 2 and 3). The third CCN gene, *nov*, was significantly up-regulated after 4 hours at transcriptional level (Table 2). An increased transcription of *cyr61* and *ctgf* genes has been shown during epithelial cell infection with *Y. enterocolitica* [13], *S. flexneri* [15], and *B. pertussis* [16]. CYR61, CTGF, and NOV have the capability to bind both fibronectin and *α*_5_ *β*_1_ integrin, similar to IGFBP1 and IGFBP3 [64–68], and are implicated in wound healing [68]. Taken together, up-regulation of these profibrotic genes indicates that intracellular *S. aureus* might affect the extracellular matrix by stimulating fibrosis and aiding in repair of the damage caused by *S. aureus* infection.

### 3.8. Cholesterol Biosynthesis

Intracellular *S. aureus* caused down-regulated expression of cholesterol biosynthesis enzyme genes, including sterol-c4-methyl oxidase-like (*sc4mol*), 3-hydroxy-3-methylglutaryl-coenzyme A reductase (*hmgcr*), hydroxysteroid (17*β*) dehydrogenase 7 (*hsd17b7*), isopentenyl-diphosphate delta isomerase (*idi1*), squalene monooxygenase (*sqle*), sterol c5-desaturase-like (*sc5dl*), farnesyl-disphosphate famesyltransferase 1 (*fdft1*), and 7-dehydrocholesterol reductase (*dhcr7*). Genes involved in regulation of cholesterol synthesis were also down-regulated. Insulin-induced gene 1 (*insig1*), encoding a membrane endoplasmic reticulum protein, was down-regulated (0.20-fold at 4 hours, 0.26-fold at 6 hours, and 0.41-fold at 8 hours) (Table 2). Acetyl CoA synthetase gene (*acas2*) and low-density lipoprotein receptor gene (*ldlr*) were also transcriptionally down-regulated (Table 2). QRT-PCR data confirmed the down-regulation of *hmgcr*, *sqle*, *dhcr7,* and *ldlr* (Table 3). Cholesterol quantification with GC-MS also showed that host cells displayed a corresponding decreased cholesterol synthesis after a challenge with intracellular *S. aureus* (Table 4). Garner et al. showed an essential role for cholesterol in the uptake of *S. typhimurium* into HeLa cells, demonstrating that the removal of cholesterol caused a greater than 90% decrease in bacterial uptake [69]. Thus, a reduction in cholesterol may be a response to limit the internalization of *S. aureus*. In addition, a decrease in cholesterol levels could limit the effects of *S. aureus* exotoxins on the host cell membrane. *S. aureus* alpha toxin, along with other pore-forming toxins from *Streptococcus* and *Clostridium* species, showed reduced activity when cholesterol levels in lipid membranes were decreased [70, 71]. A recent study showed that the golden *S. aureus* pigment, staphyloxanthin, is synthesized with the same substrates used for cholesterol biosynthesis by host cells [72]. It is unclear at present whether the effect on cholesterol biosynthesis is related to this finding; however, it is conceivable that this effect might represent a host response to affect production of this staphylococcal virulence factor.

In summary, this study demonstrates that several classes of genes in HEp-2 cells undergo changes in transcriptional expression in response to intracellular *S. aureus*. We observed that, in the first few hours of intracellular infection, epithelial cells can respond to intracellular *S. aureus* quickly by inducing early stress response (AP-1 complex) and MAPK pathways (Ras, P38), which consequently stimulate broader responses such as proinflammatory response, apoptosis, and fibrosis. Our data support the belief that the role of epithelial cells in innate immunity is not simply that of a physical barrier against invading pathogens, but it is also actively involved in the induction of more complex host defense mechanisms. Another possibility is that, as a successful pathogen, intracellular *S. aureus* might lead to host gene expression that facilitates its intracellular survival. This is consistent with induction of Ras-related cytoskeleton reorganization and the fibrosis process. Our results are also consistent with, although not definitive of, a delicate balance between effects which benefit the host and those which are more beneficial to *S. aureus*. Finally, this study showed that intracellular *S. aureus* suppressed cholesterol synthesis in epithelial cells. The consequence of this suppression on the pathogenesis of *S. aureus* is not clearly presented but might be related to recent observations regarding staphylococcal pigment production.

## Figures and Tables

**Table 1:** DNA primers used for QRT-PCR experiments.

Gene	Forward primers (5′-3′)	Reverse primers (5′-3′)
*atf3*	GATGTCCTCTGCGCTGGAAT	CCTCGGCTTTTGTGATGGA
*c-fos*	GCCCTTTGATGACTTCCTGTTC	GGAGCGGGCTGTCTCAGA
*c-jun*	GCAAAGATGGAAACGACCTTCT	GCTCTCGGACGGGAGGAA
*junB*	CTACACGACTACAAACTCCTGAAACC	CCCCAGGCGCTTTGAGA
*sgk*	GTGCCTGGGAGCTGTCTTGT	GCTGTGTTTCGGCTATAAAAAGG
*arhb*	TCCCAATGTGCCCATCATC	ATGCGGGCCAGCTCTGT
*map2k3*	CCCTACATGGCCCCTGAGA	TCCAGACGTCGGACTTGACA
*Il1b*	CGAATCTCCGACCACCACTAC	TCCATGGCCACAACAACTGA
*tnfa*	CCTGGTATGAGCCCATCTATCTG	TAGTCGGGCCGATTGATCTC
*Il6*	AGCCGCCCCACACAGA	TCGAGGATGTACCGAATTTGTTT
*Il8*	CTGGCCGTGGCTCTCTTG	CTTGGCAAAACTGCACCTTCA
*ccl20*	TCCTGGCTGCTTTGATGTCA	AAAGTTGCTTGCTGCTTCTGATT
*cxcl1*	AACATCCAAAGTGTGAACGTGAA	GAGTGTGGCTATGACTTCGGTTT
*Il10*	CTTGTCTGAGATGATCCAGTTTTACCT	CCTTGATGTCTGGGTCTTGGTT
*ptgs2*	GGAAGCCTTCTCTAACCTCTCCTATT	AGGGAGTCGGGCAATCATC
*adm*	GGATGTCGCGTCGGAGTTT	TGCTGGACATCCGCAGTTC
*dkk1*	AAGTACCAGACCATTGACAACTACCA	GGGACTAGCGCAGTACTCATCAGT
*igfbp1*	CCATCTGATGGCCCCTTCT	CCTTCGAGCCATCATAGGTACTG
*casp9*	AGGACATGCTGGCTTCGTTT	TTCTAGGGTTGGCTTCGACAA
*tgfb1*	CCTGGCGATACCTCAGCAA	CCGGTGACATCAAAAGATAACCA
*thbs1*	TCCGCAAAGTGACTGAAGAGAA	TGAACTCCGTTGTGATAGCATAGG
*cyr61*	GGTGGAGTTGACGAGAAACAATG	AGGGAGCCGCTTCAGTGA
*hmgcr*	CCCAGTTGTGCGTCTTCCA	TGCGAACCCTTCAGATGTTTC
*sqle*	CGCCCTCTTCTCGGATATTCT	CCGAGCTGCTCCTTATTTTCTG
*dhcr7*	AGCCGCCCAGCTCTATACCT	TTATGGCAGAAGTCAGGGAGAGA
*ldlr*	GATGAAGTTGGCTGCGTTAATGT	CGCCGCTGTGACACTTGA
*actb*	CGTTGCTATCCAGGCTATGCT	TCACCGGAGTCCATCACGAT

**Table 2:** Microarray analysis of gene expression changes in infected HEp-2 cell monolayers.

Category	Gene	Fold change (*P* value)			
		2 h	4 h	6 h	8 h
Stress response	*atf3*	7.89 (.024)	1.53 (.113)	2.00 (.041)	0.85 (.189)
	*c-fos*	2.59 (.052)	1.21 (.072)	0.911 (.302)	0.843 (.287)
	*fosB*	2.29 (.009)	1.14 (.605)	0.95 (.919)	1.10 (.101)
	*c-jun*	1.88 (.011)	1.40 (.094)	0.77 (.064)	1.27 (.035)
	*junB*	1.98 (.007)	1.16 (.015)	1.22 (.015)	1.03 (.407)
	*sgk*	4.17 (.018)	2.16 (.005)	1.84 (.178)	2.13 (.066)

Signal transduction	*map2k1*	1.23 (.835)	1.52 (.025)	1.80 (.030)	2.86 (.041)
	*arhe*	2.61 (.005)	2.31 (.060)	1.26 (.370)	1.70 (.085)
	*arhb*	2.21 (.044)	2.18 (.004)	1.78 (.065)	1.60 (.022)
	*ack-1*	0.67 (.061)	0.56 (.081)	0.39 (.004)	0.51 (.026)
	*map2k3*	1.98 (.014)	2.41 (.003)	1.96 (.127)	2.33 (.052)

Proinflammatory response	*cox2*	3.32 (.012)	2.71 (.088)	1.29 (.065)	2.55 (.064)

Cell proliferation and proapoptosis	*dkk1*	2.17 (.026)	7.11 (.001)	3.23 (.012)	4.52 (.055)
	*klf4*	2.33 (.001)	1.78 (.134)	1.61 (.008)	1.61 (.104)
	*klf6*	2.51 (.019)	1.50 (.064)	1.52 (.046)	1.62 (.090)
	*Igfbp1*	2.54 (.062)	4.32 (.001)	2.13 (.086)	11.10 (.030)
	*Igfbp3*	0.77 (.664)	1.80 (.051)	3.65 (.003)	2.23 (.088)
	*casp9*	1.95 (.015)	1.57 (.021)	0.54 (.617)	0.78 (.666)
	*bnip3*	1.25 (.073)	1.64 (.034)	2.86 (.002)	2.47 (.041)
	*nur77*	6.17 (.038)	1.28 (.181)	0.78 (.114)	0.87 (.235)

Profibrotic	*tgfbr2*	1.47 (.011)	1.67 (.083)	2.09 (.008)	2.10 (.010)
	*v-erb-b*	0.96 (.608)	1.67 (.055)	1.96 (.005)	2.11 (.007)
	*itga5*	0.88 (.768)	2.19 (.037)	2.12 (.033)	3.20 (.030)
	*thbs1*	1.28 (.112)	2.93 (.001)	2.54 (.024)	2.44 (.209)
	*pai1*	2.45 (.006)	1.80 (.040)	1.27 (.089)	1.32 (.136)
	*pai2*	ND	2.09 (.015)	4.43 (.001)	4.13 (.013)
	*cyr61*	4.43 (.007)	3.24 (.001)	2.33 (.152)	1.61 (.249)
	*ctgf*	6.78 (.026)	2.13 (.141)	ND	ND
	*nov*	1.46 (.301)	1.98 (.059)	2.05 (.002)	2.15 (.006)

Cholesterol synthesis	*sc4mol*	0.81 (.367)	0.32 (.001)	0.36 (.001)	0.31 (.001)
	*hmgcr*	1.11 (.700)	0.30 (.002)	0.21 (.001)	0.36 (.016)
	*hsd17b7*	0.74 (.189)	0.50 (.001)	0.30 (.006)	0.31 (.014)
	*idi1*	1.00 (.979)	0.53 (.018)	0.33 (.004)	0.31 (.151)
	*sqle*	0.90 (.397)	0.50 (.007)	0.26 (.001)	0.31 (.008)
	*sc5dl*	0.90 (.358)	0.45 (.017)	0.23 (.008)	0.35 (.064)
	*fdft1*	0.96 (.551)	0.59 (.002)	0.29 (.001)	0.26 (.010)
	*dhcr7*	0.95 (.527)	0.63 (.010)	0.51 (.016)	0.39 (.014)
	*insig1*	0.69 (.338)	0.20 (.001)	0.26 (.001)	0.41 (.014)
	*acas2*	ND	0.58 (.080)	0.27 (.001)	0.37 (.024)
	*ldlr*	1.09 (.048)	0.41 (.002)	0.54 (.017)	0.68 (.114)

ND: Not determined. Data not shown due to low signal intensity (<50).

**Table 3:** Validation of selected genes by QRT-PCR.

Category	Gene	Fold change (*P* value)			
		2 h	4 h	6 h	8 h
Stress response	*atf3*	15.45 (.001)	4.46 (.005)	1.38 (.004)	1.97 (.027)
	*c-fos*	4.55 (.001)	1.32 (.005)	1.47 (.001)	1.86 (.001)
	*c-jun*	2.93 (.001)	1.26 (.001)	1.77 (.001)	2.91 (.001)
	*junb*	3.60 (.001)	1.26 (.001)	1.37 (.008)	2.29 (.004)
	*sgk*	3.36 (.001)	1.20 (.001)	1.87 (.001)	2.02 (.001)

Signal transduction	*arhb*	2.61 (.002)	1.80 (.001)	2.17 (.001)	1.71 (.019)
	*map2k3*	1.57 (.001)	1.89 (.013)	2.04 (.005)	2.12 (.001)

Proinflammatory response	*il1b*	3.64 (.001)	1.58 (.007)	2.02 (.001)	1.47 (.002)
	*tnfa*	3.36 (.001)	1.30 (.009)	2.21 (.001)	1.43 (.001)
	*il6*	2.65 (.001)	1.87 (.001)	2.96 (.001)	1.55 (.004)
	*ccl20*	6.29 (.001)	5.07 (.002)	4.53 (.001)	1.82 (.001)
	*cxcl1*	3.82 (.001)	2.41 (.001)	2.87 (.001)	3.05 (.050)
	*cox2*	4.16 (.001)	3.69 (.001)	3.96 (.001)	2.95 (.011)

Cell proliferation and Proapoptosis	*dkk1*	3.43 (.001)	6.90 (.001)	4.31 (.001)	2.28 (.001)
	*igfbp1*	3.50 (.001)	6.82 (.045)	4.20 (.010)	9.89 (.001)
	*casp9*	2.74 (.001)	1.34 (.005)	1.42 (.004)	1.13 (.021)

Profibrotic	*tgfb1*	1.55 (.002)	1.20 (.023)	1.71 (.001)	2.66 (.001)
	*thbs1*	1.83 (.001)	4.55 (.001)	4.12 (.002)	4.09 (.001)
	*cyr61*	3.31 (.001)	4.01 (.002)	2.28 (.016)	2.44 (.001)

Cholesterol synthesis	*hmgcr*	1.25 (.025)	0.17 (.001)	0.15 (.001)	0.17 (.001)
	*sqle*	1.00 (.005)	0.30 (.001)	0.14 (.001)	0.15 (.001)
	*dhcr7*	1.17 (.050)	0.50 (.001)	0.27 (.001)	0.16 (.001)
	*ldlr*	1.57 (.010)	0.09 (.001)	0.12 (.001)	0.23 (.001)

**Table 4:** Cholesterol quantification [(*μ*g/10^5^ cells) ± SD] in uninfected and infected HEp-2 cell monolayers.

Cell type	Incubation time (h)			
	2	4	6	8
Unchallenged HEp-2 cell	75.82 ± 2.99	69.78 ± 5.31	64.94 ± 5.00	55.91 ± 4.78
Challenged HEp-2 cell	58.43 ± 3.73	51.92 ± 2.87	50.74 ± 5.22	43.38 ± 3.36
% cholesterol reduction	22.91	25.60	21.87	22.41

*P* value	.005	.050	.045	.026
